# Delivery Systems Based on Innovative Nanomaterials

**DOI:** 10.3390/nano12081296

**Published:** 2022-04-11

**Authors:** Tânia Santos de Almeida, Catarina Pereira-Leite

**Affiliations:** 1 CBIOS—Universidade Lusófona’s Research Center for Biosciences & Health Technologies, Campo Grande 376, 1749-024 Lisbon, Portugal; 2 Centro de Química Estrutural, Institute of Molecular Sciences, Departamento de Química e Bioquímica, Faculdade de Ciências, Universidade de Lisboa, Campo Grande, 1749-016 Lisbon, Portugal; 3 LAQV, REQUIMTE, Departamento de Ciências Químicas, Faculdade de Farmácia, Universidade do Porto, Rua de Jorge Viterbo Ferreira 228, 4050-313 Porto, Portugal

There has been an increasing interest in using nanomaterials to develop innovative delivery systems. This is due to the fact that nano-based delivery systems present several valuable characteristics such as the ability to move easily within the human body when compared with other larger systems, allowing for controlled drug delivery and improved drug bioavailability. Moreover, nanosystems may be optimized and functionalized when blended with other materials. Consequently, there has been a growing investment in developing new **delivery systems based on innovative nanomaterials**, which is the topic of this Special Issue.

This Special Issue encompasses a review that addresses the potential of nanocarriers for the delivery of ceramides and glucocorticoids in the topical treatment of skin disorders with barrier impairment [1]. Various skin diseases are associated with epidermal barrier disruption and alterations in the lipid composition of the stratum corneum (SC), including xerosis, ichthyosis, atopic dermatitis, and psoriasis. A general hallmark of these diseases is reduced levels of ceramides in the SC, so that their management includes ceramide-containing formulations. As a strategy to promote the skin permeation of ceramides and to favor their beneficial effects, various nanodelivery systems have been developed to deliver ceramides, such as polymeric nanoparticles, solid lipid nanoparticles, nanostructured lipid carriers, nanoemulsions, and vesicular nanosystems. Moreover, management of the abovementioned skin diseases usually depends on the topical application of glucocorticoids. These therapeutic agents display limited skin penetration and considerable adverse effects, justifying the development of polymeric nanoparticles, solid lipid nanoparticles, nanostructured lipid carriers, and hybrid nanoparticles to upgrade the efficacy and safety of the topical glucocorticoid therapy.

The use of nanocarriers in the context of oncology has been widely explored, and in this Special Issue, Faisalina et al. [2] produced nanoparticles made of poly(3-hydroxybutyrate-*co*-4-hydroxybutyrate) (P(3HB-*co*-4HB)) copolymers to deliver docetaxel, an anticancer drug for the treatment of breast, lung, hormone-refractory prostate, and advanced gastric cancer. Three different P(3HB-*co*-4HB) copolymers were tested, since this type of polyhydroxyalkanoate copolymer displays interesting in vivo degradation kinetics that can be modulated by varying the 4-hydroxybutyrate (4HB) fraction. The nanoparticles obtained displayed particle sizes between 140 and 180 nm with uniform size distributions, spherical morphologies, and smooth surfaces. The most promising formulation was produced with 70% of 4HB monomer content, showing an encapsulation efficiency of ~50% with no aggregation phenomena. By controlling the drug-polymer ratios, this nanosystem showed a controlled release profile; thus, it may be considered a potential candidate for docetaxel delivery to ultimately improve its efficacy and safety.

Rutin is a natural compound that exhibits several interesting properties, such as anti-inflammatory, antioxidant, and anticancer properties, which may be useful in the pharmaceutical field. However, the low solubility of this compound in water may impair its applicability. Within this matter, the development of innovative nanomaterials may be a strategy used to overcome this challenge to ultimately allow for the inclusion of rutin in anti-inflammatory, anti-ageing, and anticancer formulations. This issue includes two studies [3,4] that showcase this approach and cover the development of different nanosystems that incorporate rutin.

Giuliano et al. [3] reported the development and characterization of new P407-based hydrogels containing rutin, since P407 is known to promote the aqueous solubility of many compounds. The authors showed that the incorporation of rutin up to 0.1% (*w*/*w*) into the P407 solutions did not compromise the stability or the rheological properties of the formulations. It was also shown that the formulations allowed for a constant and prolonged drug leakage and that they may be suitable for in situ administration of rutin due to the versatility of these thermo-sensitive gelling systems, allowing for controlled release.

The other study concerning rutin related to the development of a new class of nanovesicular systems [4]. This study considered the fact that, even though transfersomes can be an interesting strategy in controlled delivery, particularly for cutaneous delivery, these systems may still be upgraded. In consequence, Júlio et al. developed innovative transfersomes containing ionic liquids (ILs), denominated as TransfersomILs, based on an optimized formulation that was obtained from a Box–Behnken factorial design (BBD). The TransfersomILs were prepared containing ILs and IL:IL combinations and incorporating rutin. This study showed that the TransfersomILs had a smaller particle size. Generally, these new systems also presented a higher association efficiency, loading capacity, and total amount of rutin release when compared with the transfersomes without IL. Moreover, the study showed as well that the type of IL used may also have a positive impact on the stability of the obtained formulations.

Finally, we acknowledge all the authors who contributed with their work to this Special Issue as well as to the editorial board of *Nanomaterials* for all of their support.
